# A Conserved Rule for Pancreatic Islet Organization

**DOI:** 10.1371/journal.pone.0110384

**Published:** 2014-10-28

**Authors:** Danh-Tai Hoang, Hitomi Matsunari, Masaki Nagaya, Hiroshi Nagashima, J. Michael Millis, Piotr Witkowski, Vipul Periwal, Manami Hara, Junghyo Jo

**Affiliations:** 1 Asia Pacific Center for Theoretical Physics, Pohang, Korea; 2 Meiji University International Institute for Bio-Resource Research, Kanagawa, Japan; 3 Department of Surgery, The University of Chicago, Chicago, IL, United States of America; 4 Laboratory of Biological Modeling, NIDDK, NIH, Bethesda, MD, United States of America; 5 Department of Medicine, The University of Chicago, Chicago, IL, United States of America; 6 Department of Physics, POSTECH, Pohang, Korea; NIDCR/NIH, United States of America

## Abstract

Morphogenesis, spontaneous formation of organism structure, is essential for life. In the pancreas, endocrine 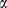, 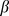, and 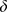 cells are clustered to form islets of Langerhans, the critical micro-organ for glucose homeostasis. The spatial organization of endocrine cells in islets looks different between species. Based on the three-dimensional positions of individual cells in islets, we computationally inferred the relative attractions between cell types, and found that the attractions between homotypic cells were slightly, but significantly, stronger than the attractions between heterotypic cells commonly in mouse, pig, and human islets. The difference between 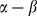 cell attraction and 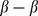 cell attraction was minimal in human islets, maximizing the plasticity of islet structures. Our result suggests that although the cellular composition and attractions of pancreatic endocrine cells are quantitatively different between species, the physical mechanism of islet morphogenesis may be evolutionarily conserved.

## Introduction

Multi-cellular organisms require communications between neighboring cells, and have developed special architectures for optimizing such cellular communications. A fundamental question in life is how organisms spontaneously form their functional structures. Interestingly, a few simple rules can be sufficient to form complex organs such as the lung [1]. As a microscopic explanation of morphogenesis, Steinberg introduced the *differential adhesion hypothesis* that differences in adhesiveness between cell types are partially responsible for the development and maintenance of organ structures [2], [3].

Pancreatic islets of Langerhans are the critical micro-organs responsible for glucose homeostasis. Each islet consists mainly of 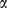, 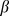, and 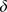 cells. Glucagon and insulin are the reciprocal hormones for increasing and decreasing blood glucose levels, secreted by 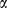 and 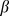 cells, respectively. The role of 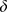 cells in glucose homeostasis is still mysterious. In addition, it has long been reported that endocrine cells interact with each other [4]. Considering the specific symmetries of interactions between 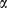, 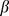, and 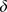 cells, their spatial organization must have functional significance. Rodent islets have a shell-core structure where 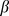 cells are located in the islet core, while non-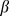 cells are located on the islet periphery. However, there are contradictory reports regarding the structure of human islets [5]. Some observations suggest more or less random structures of cells [6], [7], while others have found some order in structures, and described human islets as assemblages of 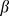-cell-core subunits [8] or lobules [9], cloverleaf patterns [5], ribbon-like structures [10], and folded trilaminar plate [11].

Dissociated islet cells spontaneously aggregate and form islet-like structures, *pseudo-islets*, in rat, pig, and human pancreatic cultures [12]–[15]. Different adhesion molecules have been proposed as a cause of the pseudo-islet formation expressed on rodent 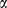 and 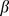 cells [16]–[20]. However, the relative adhesion strengths of such cells in native islets has not been directly measured. On one hand, this technical limitation leaves open the interesting question of whether different species have different rules for islet organization. On the other hand, current imaging methods allow to observe islet structures with high resolution. In this study, we computationally infer the organization rule from three-dimensional islet structures. In particular, we compare mouse, pig, and human islets, and find a conserved organization rule behind different islet structures.

## Results

### Cellular distributions in pancreatic islets

**Figure 1:**
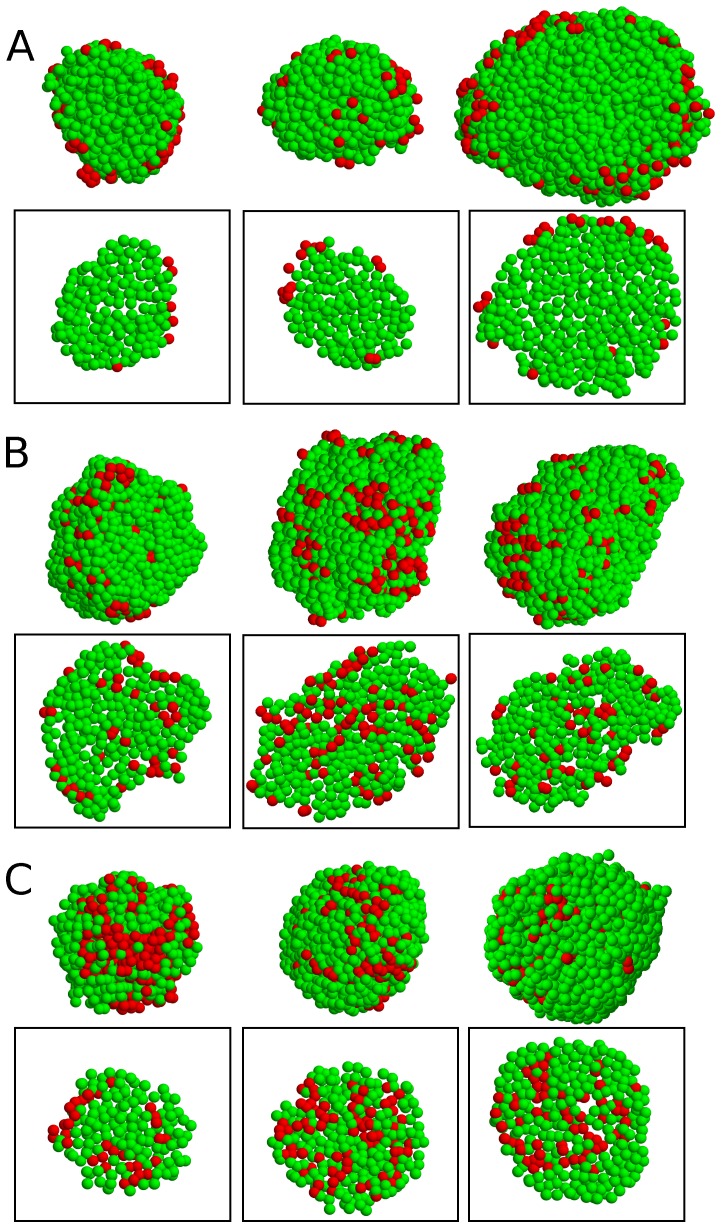
Cellular organization of pancreatic islets. Three-dimensional spatial distribution of *α* cells (red) and *β* cells (green) is shown in (*A*) mouse, (*B*) pig, and (C) human islets. To show internal islet structures clearly, their corresponding two-dimensional sections are also shown in boxes.

Two-dimensional cross sections of pancreatic islets have been widely used to study islet structures. Here, however, we used a confocal microscope to examine three-dimensional islet structures, as previously used [6], [21]. To precisely analyze islet structures, we obtained three-dimensional positions of every endocrine cell in individual islets. We used stained nuclei to determine the center positions of cells, and immunostaining to determine cell types. Figure 1 showed the spatial cellular organizations of pancreatic islets in different species. First, we observed two major cell types of 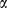 and 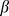 cells in mouse (Fig. 1*A*), pig (Fig. 1*B*), and human islets (Fig. 1*C*). Mouse islets showed the typical shell-core structure in which 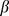 cells are located in the core, while 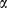 cells are located on the periphery. In pig and human islets, however, 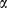 cells are located not only on the periphery but also distributed inside islets.

**Figure 2:**
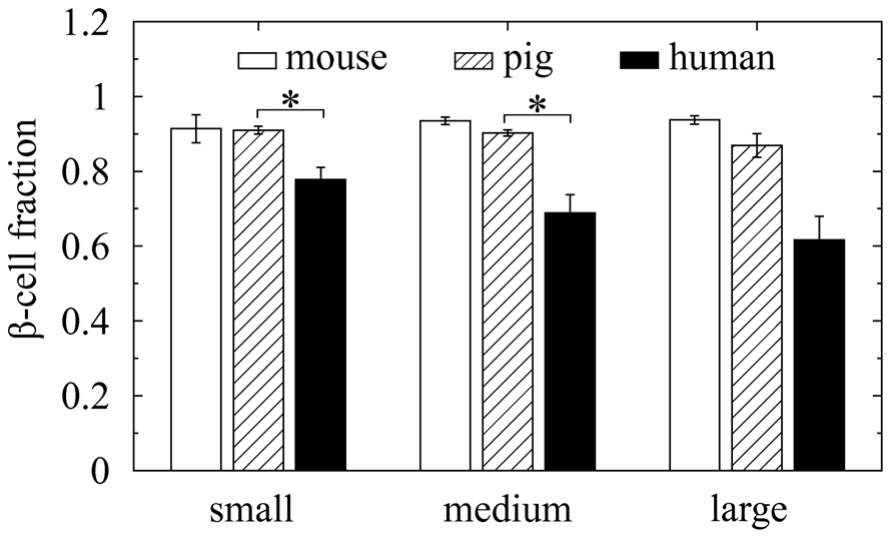
Cellular compositions in mouse, pig, and human islets. Fractions of *β* cells, depending on islets size, are calculated in mouse (empty bar), pig (hatched), and human (black solid) islets. Islet size is represented by the total number of cells in islets, and categorized as small (<1000 cells), medium (1000–2000), and large (>2000) islets. Mean ± SEM (n = 30). ^*^ *P*<0.005.

**Figure 3:**
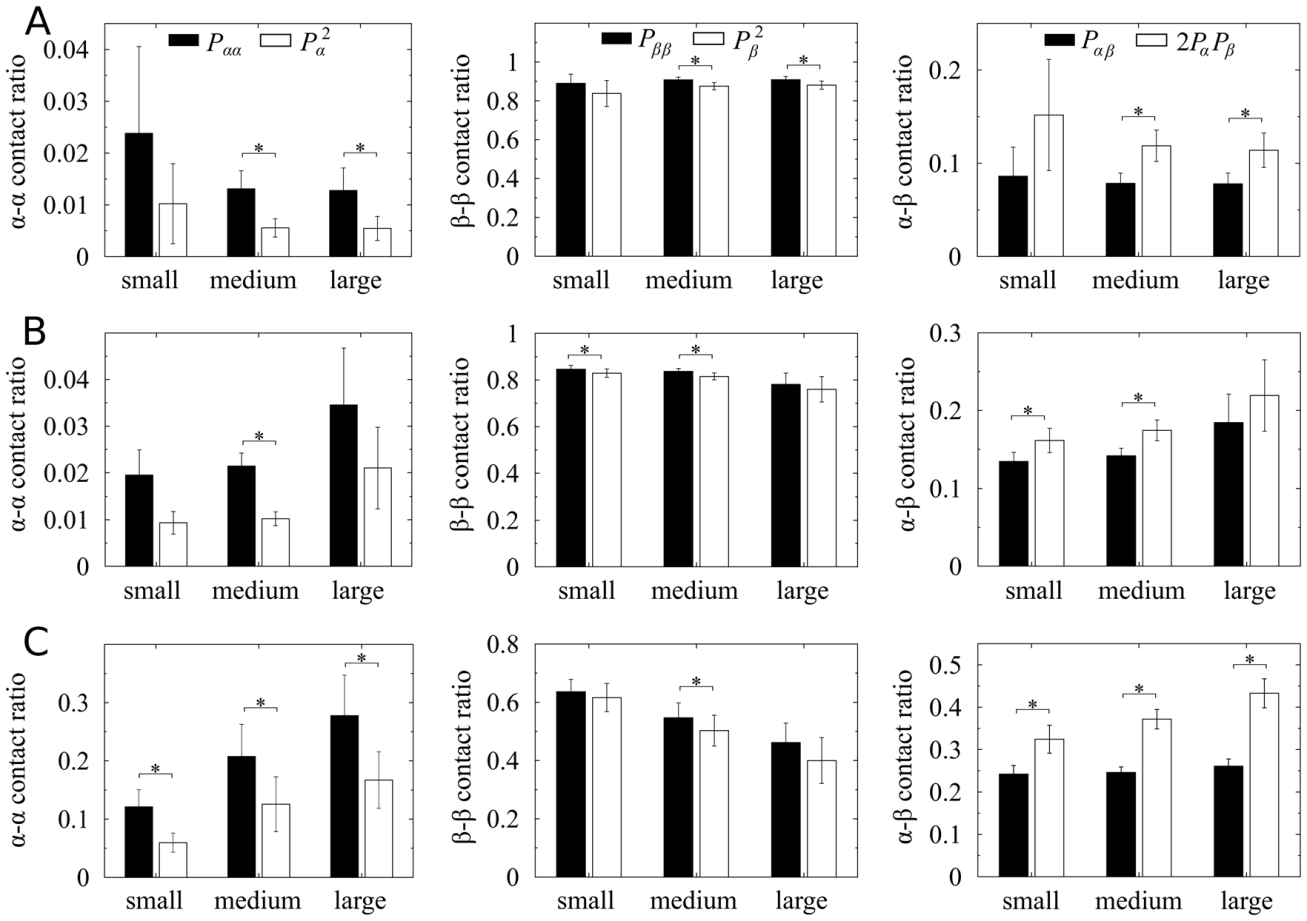
Cell-to-cell contact ratios in mouse, pig, and human islets. Based on the contacts between neighboring cells, ratios of 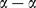, 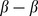, and 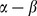 contacts (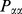, 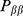, and 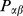), depending on islet size, are calculated in (*A*) mouse, (*B*) pig, and (*C*) human islets. Islet size is represented by the total number of cells in islets, and categorized as small (<1000 cells), medium (1000–2000), and large (>2000) islets. Given fractions of 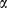 and 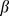 cells (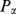 and 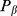), the 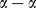, 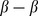, and 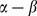 contact probabilities in random cell organization are theoretically 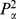, 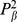, and 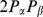, respectively. The random organization (empty bar) is compared with the organization of native islets (black solid). Mean ± SEM. ^*^ *P*<0.005.

We then quantified the fraction of 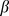 cells depending on islet size in the three species (Fig. 2). Mouse islets consisted of 90% 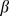 cells independent of islet size. Human islets had a smaller 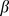-cell fraction, 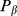. In particular, larger human islets had less abundant 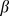 cells depending on size. This finding in three-dimensional islets is consistent with previous reports based on pancreatic sections [11], [22], [23]. Interestingly, pig islets (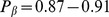) showed an intermediate characteristic between mouse (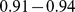) and human islets (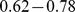). We also examined cell-to-cell contacts (See Materials and Methods), and quantified their ratios, 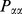, 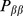, and 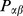 for 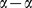, 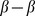, and 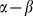 contacts, respectively (Fig. 3). The higher 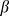-cell fraction in mouse islets resulted in more prevalent 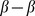 contacts (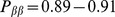), compared with pig (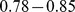) and human islets (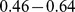), but less prevalent 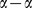 and 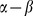 contacts.

Given fractions of 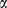 and 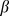 cells, we could simulate cell-to-cell contact probabilities in random cell aggregates. The probability that two sites are occupied randomly by 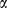 cells is 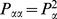, and the one for 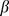 cells is 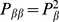. In addition, the probability that two sites are occupied randomly by 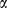 and 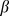, or vice versa, is 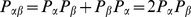. Regardless of species, Fig. 3 shows that frequencies of homotypic contacts are significantly higher than the probabilities in random aggregates (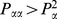 and 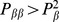). On the other hand, islet structures showed smaller frequencies of heterotypic contacts, compared with random (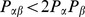). These results clearly demonstrated that islet structures are not random cell aggregates. This conclusion looks trivial for the shell-core structure of mouse islets (Fig. 1*A*). However, this suggested that pig and human islets also had some order in their cellular organization.

### Self-organization rule for pancreatic islets

**Figure 4:**
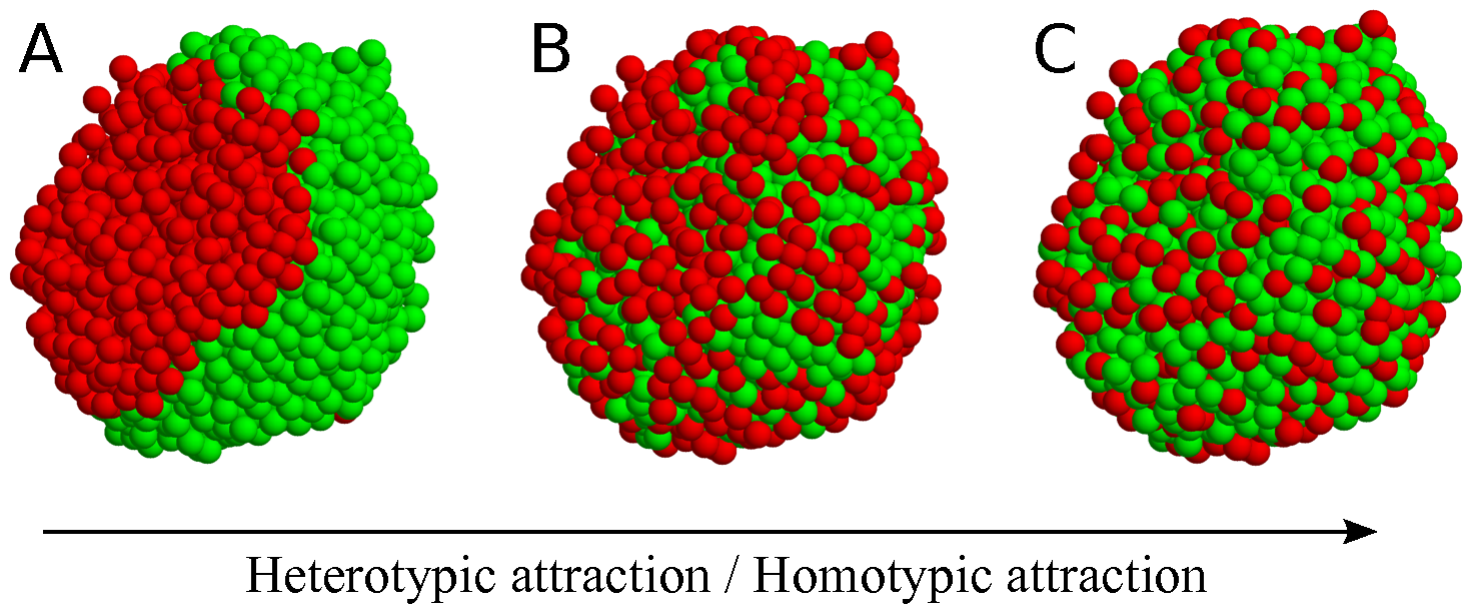
Schematic diagram of structural dependence on relative attractions between cell types. A sorting structure of two cell types is changed to mixing structures, as heterotypic attraction increased compared with homotypic attractions: (*A*) complete sorting, (*B*) shell-core sorting, and (*C*) mixing structures.

Since islets are not random cell aggregates, we investigated rules governing islet structure. Prevalent contacts of homotypic cells could have resulted from (i) replication of neighboring cells and/or (ii) stronger attraction between homotypic cells. Islet organogenesis occurs in the milieu of developmental processes including cell differentiation, migration, aggregation, replication, and death [24], [25]. Nevertheless, when islet cells are dissociated, they can spontaneously form pseudo-islets resembling native islets [12]–[15]. This pseudo-islet formation implies that sequences of complicated developmental processes, particularly cell replication, may not be critical for the formation of equilibrium islet structures. Therefore, as proposed [2], [3], [26], we investigated if the differential adhesion hypothesis could provide a simple rule governing for islet structures. Depending on the relative adhesiveness between cell types, islets could have various equilibrium structures (Fig. 4). When homotypic attractions are stronger than heterotypic attractions, islets have a sorting phase with two homogeneous cell clusters. As heterotypic attraction becomes stronger, the two cell types start to mix [27].

We specify the relative strengths of adhesiveness or attraction between cell types as 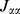, 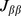, and 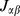 for 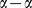, 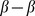, and 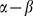 contacts, respectively. A stronger attraction between neighboring cells implies that it requires a larger amount of energy to dissociate them. Therefore, the total cell-to-cell contact energy, *self-energy*, in an islet is a sum over every contact
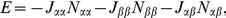
(1)where the islet has total 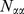, 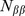, and 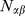 contacts of 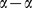, 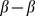, and 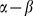, respectively. The negative sign in Eq. (1) represents that external energy is needed (not extracted) to dissociate cell-to-cell contacts. Given numbers of 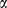 and 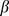 cells, the islet self-energy can have various values depending on spatial organization of cells. Our conjecture is that islets have an equilibrium structure that minimizes their self-energy. When homotypic attractions are stronger, 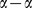 and 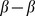 contacts are preferred. On the other hand, when heterotypic attraction is stronger, 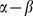 contacts are preferred.

**Figure 5:**
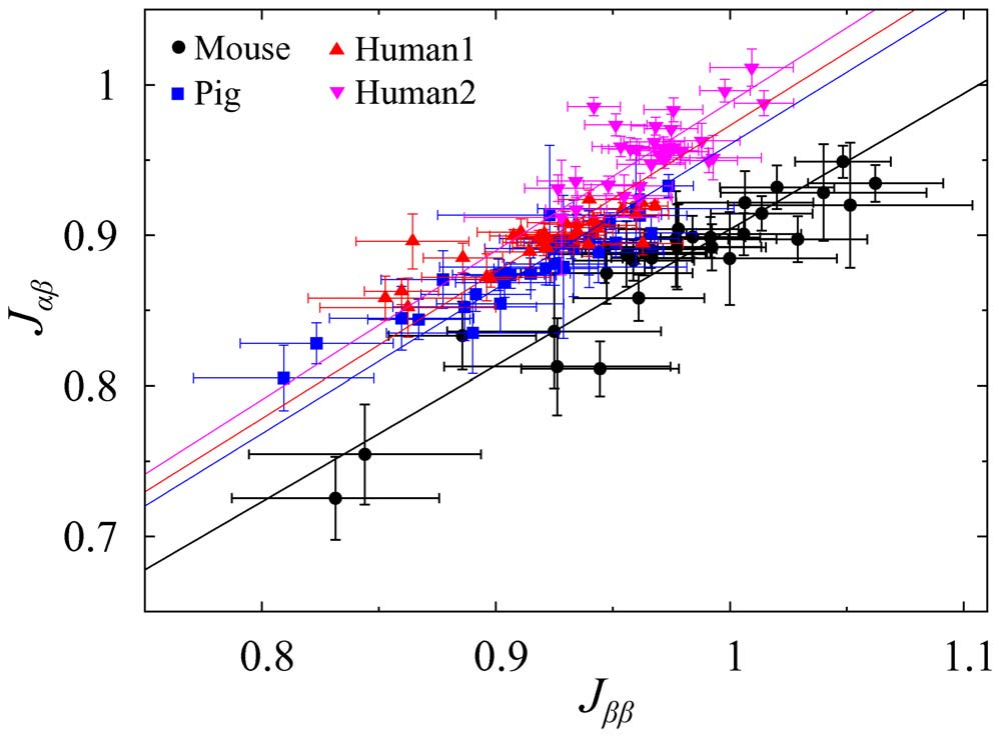
Cellular attractions in mouse, pig, and human islets. Relative attractions between cell types and their uncertainties are inferred from three-dimensional islet structures. Symbols represent individual islets: mouse (black circle), pig (blue square), and human islets (red triangle and pink inverse triangle). Each species has n = 30 islets. In particular, two sets of n = 30 islets are provided from two human (Human1 and Human2) subjects. The relationship between 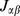 and 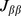 is fitted with linear functions, 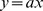, represented by solid lines with colors corresponding to each species. Note that the attraction between 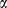 cells is defined as a reference attraction, 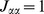.

Here our problem is not to obtain equilibrium structures given cellular attractions. It is currently not possible to measure the strengths of cellular attractions inside islets. However, we could obtain cell-to-cell contact information from three-dimensional islet imaging. Thus we addressed this inverse problem to infer the strengths of cellular attractions from cell-to-cell contact information. Using Bayesian inference (See Materials and Methods), we inferred the likelihood strengths of cellular attractions that explain observed islet structures. Figure 5 showed the inferred attractions, 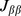 and 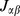, relative to the reference attraction 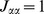, from mouse, pig, and human islets. The relative attractions were not dependent on islet size for all species (Fig. S1). Their averages and standard deviations are summarized in Table 1. The relative attractions were not dramatically different between each other, regardless of species. The homotypic attractions, 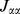 and 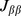, were slightly, but significantly, larger than the heterotypic attraction, 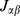, for all species considered. This general conclusion is consistent with the cellular organization, 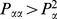, 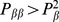, and 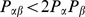. A random organization can be obtained with equal cellular attractions, i.e., 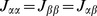. It is thus understandable that islets have 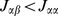 and 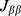. However, the Bayesian inference quantified the dependence of relative cellular attractions on species: 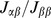 for mouse islets (0.91) is lowest, while human islets (0.98 and 0.99 for two different pancreata) show the largest ratio. Pig islets showed a similar ratio (0.97) to human islets.

**Table 1:** Cellular attractions in mouse, pig, and human islets.

Species	n	*J* *_ββ_*	*J_αβ_*	*J_αβ_*/*J_ββ_*
Mouse	30	0.97±0.05	0.88±0.05^a^	0.91±0.02
Pig	30	0.91±0.04	0.88±0.03^a^	0.97±0.02^b^
Human1	30	0.92±0.03	0.90±0.02^a^	0.98±0.02
Human2	30	0.97±0.02	0.96±0.02^a^	0.99±0.02

Relative attractions between cell types are inferred from three-dimensional islet structures, mean ± SD (n = 30 islets). Note that the attraction between 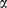 cells is defined as a reference attraction, 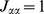.

a Paired Student's t-test concludes *J_ββ_*>*J_αβ_* with 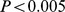.

b Unpaired Student's t-test concludes that mouse and pig islets have different *J_αβ_*/*J_ββ_* with 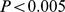.

**Figure 6:**
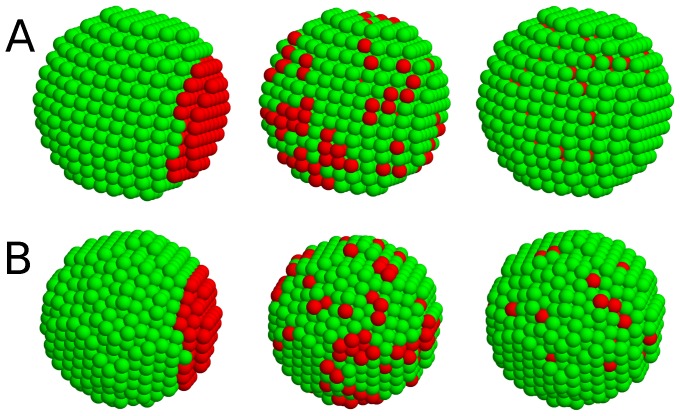
Distinct structures of binary mixtures. Complete sorting, shell-core sorting, and partial mixing structures are plotted for (*A*) cubic and (*B*) hexagonal close packed lattices. Here each lattice consists of 1357 cells with 10% 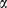 cells (red) and 90% 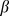 cells (green). The relative attractions are chosen to have the specific structures: 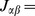
0.7 (left), 0.85 (middle), and 1.1 (right) for (*A*) the cubic lattice; and 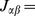
0.7 (left), 0.93 (middle), and 1.1 (right) for (*B*) the hexagonal close packed lattice. Note that the homotypic attractions are fixed as a reference, 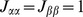, and the thermal fluctuation energy is 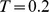.

**Figure 7:**
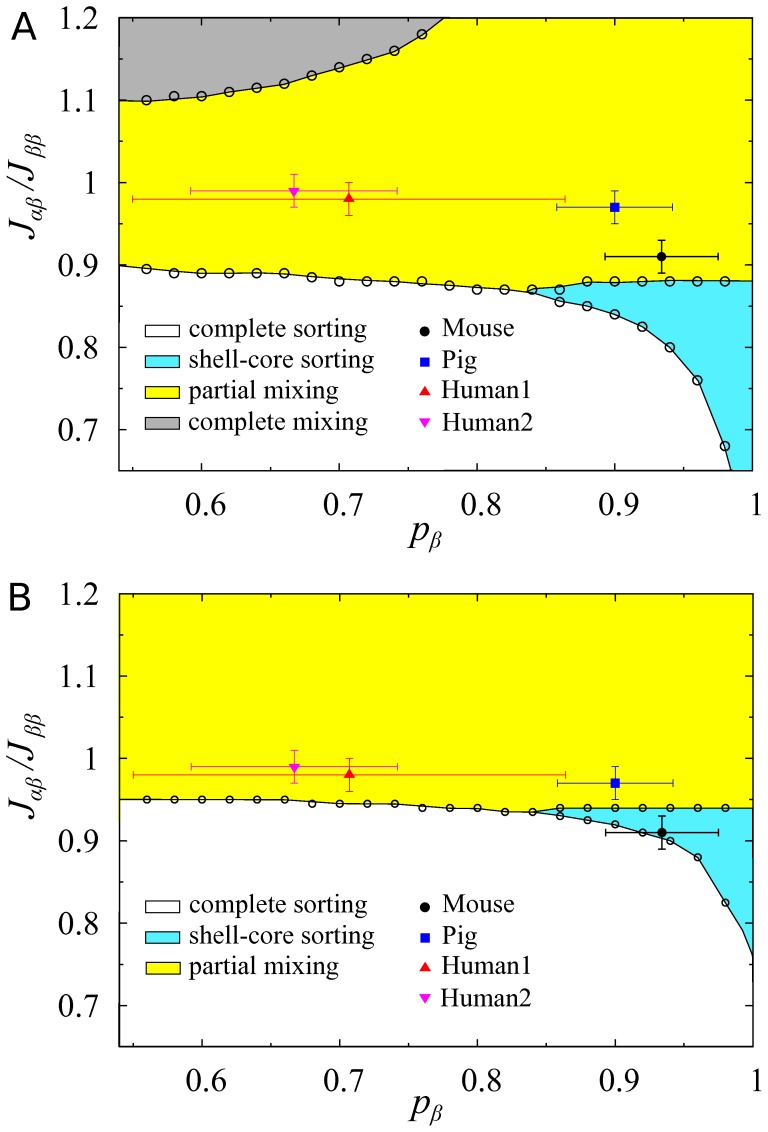
Phase diagrams of binary mixtures. Binary mixtures have complete sorting (white region), shell-core sorting (cyan region), partial mixing (yellow region), and complete sorting (gray region) structures depending on mixture fraction and relative adhesion strengths. Plotted are phase diagrams for (A) cubic and (B) hexagonal close packed lattices with 1357 cells. Symbols represent the observed *β*-cell fraction 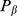 and the inferred relative attraction 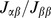 of mouse islets (black circle), pig (blue square), and human islets (red triangle and pink inverse triangle). Note that the homotypic attractions have a reference attraction, 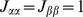. Each species has n = 30 islets. Mean ± SD.

Here, an intriguing question is whether the small differences in cellular attractions can explain the structural difference between mouse and human islets. In general, binary mixture systems of finite size can generate sorting and mixing phases depending on mixture fraction and relative adhesion strengths [27]. To answer the question, we considered two theoretical lattices representing islet structures: cubic (Fig. 6*A*) and hexagonal close packed (HCP) lattices (Fig. 6*B*), in which 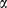 and 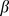 cells are distributed with various *β*-cell fractions 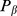 and relative attraction strengths 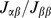. Then we computed cell-to-cell contact numbers and their fluctuations, and obtained the phase diagrams for the cubic lattice (Fig. 7*A*) and the HCP lattice (Fig. 7*B*). Human and pig islets were classified to have the partial mixing structure for both cubic and HCP lattices. However, mouse islets were classified to have the partial mixing structure for the cubic lattice, but to have the shell-core sorting structure for the HCP lattice. The native islet structure could be approximated better as the HCP lattice than the cubic lattice, because its mean number of neighboring cells (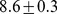 for mouse, 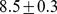 for pig, 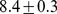 for Human1, and 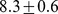 for Human2 islets) was closer to the HCP lattice (10.6) than the cubic lattice (5.4). Therefore, we concluded that the higher *β*-cell fraction 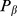 and the slightly weaker heterotypic attraction 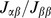 of mouse islets could be sufficient to generate the shell-core sorting structure, distinct from the partial mixing structure of human and pig islets.

### Spatial organization of δ cells

**Figure 8:**
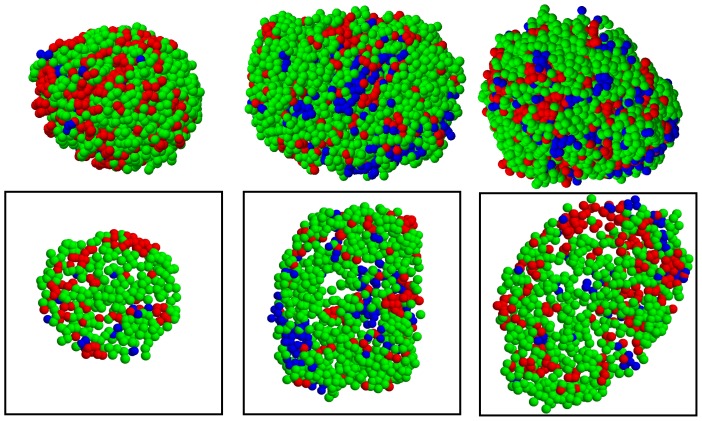
Cellular organization of human pancreatic islets. Three-dimensional spatial distribution of 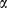 cells (red), 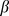 cells (green), and 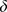 cells (blue) in human islets is shown. To show internal islet structures clearly, their corresponding two-dimensional sections are also shown in boxes. Note that islets are isolated from the Human3 subject for this plot.

In addition to 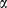 and 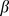 cells, islets contain a minor population of somatostatin-secreting 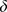 cells. We further examined human islet structures including 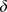 cells (Fig. 8). Similarly to previous analysis, we inferred cellular attractions, relative to 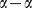 attraction (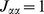), not only 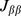 and 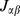, but also 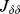, 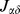, and 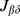 (Table 2). The result could be summarized as 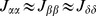 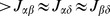. In general, homotypic attractions were slightly stronger than heterotypic attractions.

**Table 2:** Cellular compositions and attractions in human islets.

No.	Size	*P_α_*	*P_β_*	*P_δ_*	*J_ββ_*	*J_αβ_*	*J_δδ_*	*J_αδ_*	*J_βδ_*
1	588	0.25	0.54	0.21	0.97±0.02(0.96)	0.92±0.01(0.91)	1.03±0.02	0.93±0.02	0.90±0.02
2	2096	0.31	0.56	0.13	1.00±0.01(1.00)	0.94±0.01(0.93)	1.01±0.02	0.90±0.01	0.90±0.02
3	2264	0.19	0.65	0.16	0.96±0.01(0.97)	0.91±0.01(0.91)	1.00±0.02	0.90±0.01	0.89±0.02
4	2858	0.29	0.48	0.23	0.97±0.01(0.99)	0.92±0.004(0.93)	0.99±0.01	0.93±0.01	0.89±0.01
5	3253	0.34	0.47	0.19	0.98±0.01(0.99)	0.93±0.005(0.93)	1.00±0.01	0.91±0.01	0.89±0.01
6	3544	0.27	0.63	0.10	0.97±0.01(0.97)	0.92±0.005(0.92)	1.03±0.03	0.93±0.02	0.91±0.02
Av.	2434	0.28	0.55	0.17	0.98±0.01(0.98)	0.92±0.01(0.92)	1.01±0.02	0.92±0.02	0.90±0.01

Fractions of 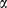, 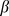, and 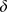 cells in six human islets, isolated from the Human3 subject, are calculated. Islet size is represented by the total number of cells in islets. Relative attractions between cell types are inferred from three-dimensional islet structures, mean ± SD. Note that the attraction between 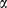 cells is defined as a reference attraction, 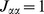. The values 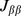 and 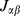 in parentheses are inferred from the three-dimensional islet structures ignoring 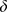 cells as empty sites.

To validate our previous analysis with islets in which 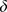 cells are unseen, we inferred 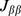 and 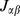 from islets in which 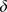 cells are seen, but ignored as empty sites. Their inferred values were not different regardless of the presence and absence of 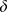 cells (Table 2).

## Discussion

We observed spatial distributions of endocrine cells in three-dimensional islets, and characterized their distributions in mouse, pig, and human islets. Islets from different species showed different cellular compositions and structures. An intriguing question was whether the structural difference originates from the different cellular composition or different organization rules, or both. Based on our computational inference from the high-resolution islet structures, we found that the adhesions between homotypic cells were slightly, but signficantly, stronger than the ones between heterotypic cells commonly in the three species. Furthermore, the binary mixture simulation on the HCP lattice demonstrated that the small difference of relative adhesions and the more abundant *β* cells could generate the shell-core structure of mouse islets, which was different from the partial mixing structure of pig and human islets. Therefore, the conserved rule could explain the different islet organizations of the three species.

We considered islet organogenesis as an equilibrium process assuming that given numbers of cells can switch their positions and minimize their total contact energy, the islet self-energy. One might consider it as a non-equilibrium process where the sequential events of cell differentiation and replication elaborately construct the specific structures of islets during development. However, sequential development is limited to explain the following two observations. First, cell replication could explain the preferential neighboring of homotypic cells, but it could not explain the regional segregation of 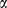 cells and *β* cells in mouse islets without extra processes such as cell polarization, migration, and death. In contrast, the equilibrium process, based on the differential adhesion hypothesis, may provide a simpler explanation for the regional segregation problem. Second, when endocrine cells are dissociated from mature islets, they can re-aggregate and form pseudo-islets resembling the native islets [12]–[15]. The pseudo-islet formation gives direct evidence suggesting that the sequence of developmental events might not be critical for the determination of islet structures. Assuming that cellular motility is sufficiently large, the detailed history of cell additions through differentiation and replication may not significantly affect the equilibrium islet structures.

Here we proposed a *dynamic structure* of islets, balancing cell motility and adhesion, instead of a static structure where cellular positions were frozen. Lymphocyte homing is an extreme example of a dynamic structure because highly mobile immune cells can organize lymphoid organs such as germinal centers and Peyer's patches through chemotoxis and adhesion [28], [29]. In this study, we quantified the cellular attraction 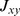 as a required energy to dissociate the contact of 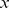 and 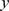 cells, and represented the cell motility 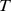 as a kind of fluctuation energy to help the cellular contacts dissociate. As the cell motility 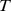 increased, cells could break their contacts to neighboring cells more frequently and move more actively. Our analysis showed that the the relative attractions between cell types were not dramatically different in pancreatic islets. Quantitatively, the energy gap between the relative cellular attractions did not exceed the fluctuation energy for cell motility to dissociate the cellular contacts, 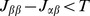. Thus islet structures become rather different from random cell organization to have a few more contacts between homotypic cells. The fine balancing of cell adhesion and motility may allow islets to have flexible structures. In particular, human islets had the minimal energy gap, 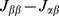, which could maximize the structural plasticity of islets. Note that this might explain the prima facie contradictory observations of human islet structure, random versus ordered structures [5]. The morphogenetic plasticity of islets has been observed [30], and proposed to have a functional implication under altered physiological conditions [31]. The islet morphology of diabetic (*db/db*) and pregnant mice shows the partial mixing structure of human islets instead of the typical shell-core sorting structure of rodent islets [31].

The differential adhesiveness between islet cells may originate from the differential expression of adhesion molecules on their cell membranes. Indeed the differential expression of neural cell adhesion molecule (NCAM) between *β* and non-*β* cells has been observed in rodent islets [17]. Furthermore, to quantify the cellular adhesiveness, the cohesivity of spherical aggregates of islet cell lines has been measured [20]. Nevertheless, it still remains to examine the expression levels of different adhesion molecules on *α*, *β*, and 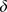 cells in different species, and find the correlation between their relative expressions and the physical attractions between cell types. In addition to the experimental demonstration of the conserved rule for the islet organization, its physiological reason remains to investigate. The coupling between *β* cells through gap junctions has been emphasized to pronounce insulin secretion [32]. Furthermore, the functional roles of paracrine interactions between *α*, *β*, and 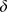 cells have been recently investigated [33], [34]. The slight preference of 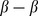 contacts to 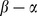 and 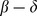 contacts may allow islets to have both advantages of *β*-cell coupling and paracrine interactions.

## Materials and Methods

### Ethics Statement

Mouse studies were approved by the Institutional Animal Care and Use Committee of the University of Chicago. Pig studies were approved by the Institutional Animal Care and Use Committee of Meiji University. The use of human tissues in the study was approved by the Institutional Review Board at the University of Chicago.

### Mouse, pig, and human islets

Mouse islets were isolated from a 3-mo old male mouse on CD-1 background. Pig islets were isolated from a 4.5-mo old female pig (a crossbred of Large white/Landrace and Duroc). Human pancreata (Human1: 60-year-old male and BMI 39.5; Human2: 40-year-old female and BMI 53; Human3: 51-year-old female and BMI 29.3) were generously provided by the Gift of Hope Organ and Tissue Donor Network in Chicago. Written informed consent from a donor or the next of kin was obtained for use of a sample in research. Specimens were collected within 12 hours of cold ischemia.

### Immunohistochemistry

Isolated islets were stained with the following primary antibodies (all 1∶500): polyclonal guinea pig anti-porcine insulin (DAKO, Carpinteria, CA), mouse monoclonal anti-human glucagon (Sigma-Aldrich, St. Louis, MO), polyclonal goat anti-somatostatin (Santa Cruz, Santa Cruz, CA), and DAPI (Invitrogen, Carlsbad, CA). The primary antibodies were detected using a combination of DyLight 488, 549, and 649-conjugated secondary antibodies (1∶200, Jackson ImmunoResearch Laboratory, West Grove, PA).

### Confocal microscopy

Microscopic images were taken with an Olympus IX8 DSU spinning disk confocal microscope (Melville, NY). To obtain coordinates of each islet cell, we manually scanned XYZ directions of a given islet, identified DAPI-stained nuclei, and determined corresponding cell types via endocrine hormone staining (i.e., insulin, glucagon, or somatostatin).

### Determination of cellular contacts

Neighboring contacts between cells were determined based on intercellular distance and angle. Note that the neighbor determination is not trivial in soft tissues unlike regular lattices. For each cell, we defined a set of cells located within a threshold distance 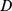 as its neighbors. Then, to discriminate some neighbors which are located close but not contacting, we measured intercellular angles between the vectors from a given vertex of each cell to its neighbors (Fig. S2). The neighbors forming narrow intercellular angles could be second-nearest neighbors. Therefore we removed those neighbors forming angles below a threshold angle 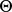 from the neighbor set of each cell. The double criteria could exclude potential errors for determining neighboring cells in large islets perhaps slightly flattened due to gravity. In practice, the mean distance between nearest nuclei can be approximated as cell diameter 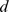. In a cubic lattice (having 6 nearest neighbors) and a hexagonal close packed lattice (12 nearest neighbors), the distances to second-nearest neighbors are 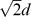 and 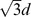, respectively. In addition, their intercellular angles between nearest neighbors are 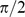 and 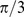. These values could guide upper limits for 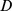 and 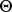. We optimized these thresholds 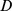 and 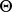 to obtain a reasonable neighboring number distribution (8 to 9 neighbors on average, 12 neighbors at most, and 1 neighbor at least). We have checked that our results were not sensitive to the thresholds.

### Model equilibration

The islet self-energy based on cellular attractions is
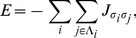
(2)where 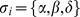 represents the cell type at the *i*th site, 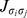 denotes the relative attractions between 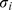 and 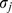, and 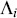 represents nearest neighbors of the *i*th site. Given cellular coordinates in an islet structure, we minimized its self-energy by exchanging positions of cells. Monte-Carlo simulation was used for the equilibration of islet structures for given cellular attractions, 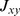 with *x*,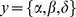 [27]. We started from measured islet structures where cell positions and types were specified. Briefly, we (i) randomly distributed given numbers of 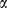, 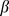, and 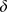 cells at the given cell coordinates in the islet; (ii) randomly chose two cells to swap, and calculated islet self-energies of 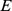 and 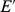 before and after exchanging their positions; (iii) accepted the exchange with the probability, 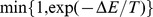, where 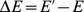 and 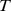 denotes thermal fluctuation, following the Metropolis algorithm [35]; and (iv) repeated these procedures in several million Monte-Carlo steps per cell to obtain an equilibrium islet structure for given cellular attractions. After equilibration, we recorded cell-to-cell contact numbers 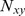 during another million Monte-Carlo steps, and calculated their mean 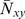 and variance 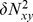. Note that this procedure is basically the same for considering the islets having only 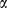 and 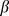 cells by ignoring 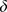 cells.

### Bayesian inference of cellular attractions

Our aim is not to obtain an equilibrium structure given cellular attraction energies, but to infer these energies given an observed structure. The basic idea is to search enumerated sets of cellular attractions, 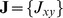 with 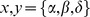, and find the likelihood sets that can explain the observed structure (Fig. S3). Summarizing, we (i) generated a random set of 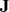; (ii) obtained an equilibrium structure given 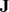; (iii) calculated the mean 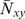 and the variance 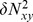 of cell-to-cell contact numbers between 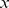 and 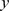 cell types for the equilibrium structure, as explained in the previous section; (iv) compared the equilibrium structure with the observed structure in terms of cell-to-cell contact numbers by quantifying their mismatches:
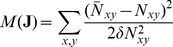
(3)where 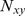 are the cell-to-cell contact numbers in the observed islet structures; and (v) repeated these procedures with a new set of 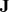 for several tens of thousands of trials. The likelihood of 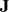 for the observed cell-to-cell contact numbers, 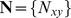, becomes
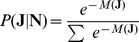
(4)thanks to the maximum entropy principle [36]. Here the summation represents all the trials of 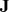 sets. Then, given all the trials or ensemble of 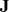, we finally could infer the mean 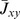 and the variance 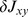 of cellular attractions for the observed islet structure:
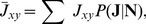
(5)

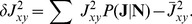
(6)


In order to optimize the calculation time, we choose 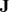 in a focus zone where the mismatch value 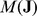 is not so large, instead of generating 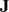 at random. For the simulation, the relative attractions 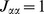 is used as unit of energy, and the fluctuation energy, which is a determinant of cell motility, is chosen large enough to escape local minimum of self-energy landscape for 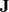, but low enough not to exceed cellular attraction energies. In particular, the fluctuation energy should be clearly less than the cellular attraction energies (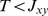). Otherwise, too large motility could detach cells from their aggregates under free boundary conditions. Therefore, we chose a reasonable fluctuation energy 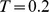. However, our conclusion was robust to different fluctuation energies (e.g., 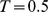; Table S1; Fig. S4).

### Phase diagram of binary mixtures

Pancreatic islets could be considered as a binary mixture of 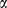 and 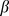 cells, the two dominant populations. In general, binary mixtures in finite systems have sorting and mixing phases depending on their composition and relative adhesion strengths between cell types. As the ratio of heterotypic attraction to homotypic attraction increases, the binary mixture has four distinct structures: complete sorting, shell-core sorting, partial mixing, and complete mixing phases [27]. We computed cell-to-cell contact numbers and their fluctuations with various *β*-cell fractions and relative attraction strengths for 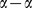, 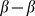, and 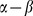 contacts. The contact number fluctuations were peaked at the boundaries between the distinct phases (Fig. S5), representing phase transitions. Based on the fluctuations, we could identify distinct structures of the binary mixture (Fig. 6), and obtained their phase diagram. For the theoretical phase diagram, we used both cubic and HCP lattices. The lattice size was fixed to have 1357 cells for both lattices. Note that the phase diagrams were not sensitive to the lattice size within the range of islet size.

## Supporting Information

Figure S1
**Relative cellular attractions and islet size.** Relative attractions between cell types are inferred from three-dimensional islet structures in (*A*) mouse, (*B*) pig, and two human, (*C*) Human1 and (*D*) Human2, subjects. Islet size is represented by the total number of cells in islets. Symbols represent individual islets, and lines represent linear data fits (dotted blue). Here the linear regression analysis rejects the null hypothesis that the relative cellular attractions depend on islet size with high 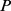 values for mouse (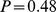), pig (0.87), Human1 (0.03), and Human2 (0.20). 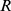 values in the plots represent the coefficient of determination. Note that the attraction between 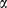 cells is defined as a reference attraction, 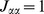. Here thermal fluctuation energy is 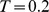.(TIF)

Figure S2
**Determination of cellular contacts.** Neighboring cells that contact to a given cell (cyan) are determined by cellular distance 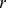 and angle 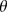. Sometimes second-nearest neighboring cells can be located within a threshold distance 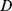 for determining neighbors. In the diagram, nearest-neighboring (red) and second-nearest neighboring (green) cells are defined as neighbors just based on distance (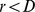). However, once we include them, intercellular angles (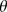 = 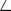
AOB) between the neighbors can be smaller than an angle threshold 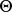. The angle threshold (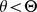) can be used to further discriminate neighboring cells, close to the given cell (cyan), but not contacting.(TIF)

Figure S3
**Flow chart for model equilibration and Bayesian inference.** We generate cellular attraction energies, 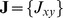 with 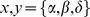, which are parameters of the differential adhesion model. Then, by using Monte-Carlo simulation, we equilibrate the islet self-energy, 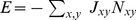, given 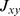, where 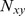 are the contact numbers between 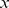 and 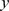 cells. After equilibration, we obtain average numbers of cellular contacts 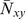, and their fluctuations 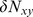. Finally, we compute the mismatch between predicted cellular contact numbers and the measured ones, 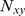. By repeating this procedure, we can have likelihood distribution of 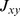, given cellular contacts **N** = 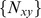. This allows to estimate the likelihood 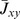 and its uncertainty 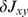.(TIF)

Figure S4
**Cellular attractions at high thermal fluctuations.** Relative attractions between cell types and their uncertainties are inferred from three-dimensional islet structures. Symbols represent individual islets: mouse (black circle), pig (blue square), and human islets (red triangle and pink inverse triangle). Each species has n = 30 islets. In particular, two sets of n = 30 islets are provided from two human (Human1 and Human2) subjects. The relationship between 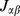 and 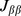 is fitted with linear functions, 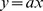, represented by solid lines with colors corresponding to each species. Note that the attraction between 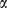 cells is defined as a reference attraction, 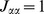. Here thermal fluctuation energy is 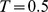.(TIF)

Figure S5
**Cell-to-cell contact number fluctuations.** The binary mixture of 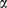 cells (40%) and 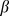 cells (60%) generates different structures depending on the relative attractions between cell types. For this plot, we fix the homotypic attractions as a reference, 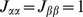, and vary the heterotypic attraction 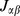. Note that the thermal fluctuation energy is 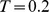. At equilibrium, the contact numbers between cell types have fluctuations, which are peaked at the boundaries between distinct structures. Here we use (*A*) cubic and (*B*) hexagonal close packed lattices with 1357 cells.(TIF)

Table S1 **Cellular attractions at high thermal fluctuations.**
(PDF)
