# Supplementary figures and images for: A Conserved Rule for Pancreatic Islet Organization
Source: PLoS One. 2014 Oct 28;9(10):e110384. doi: 10.1371/journal.pone.0110384 (PMC4211668; doi:10.1371/journal.pone.0110384)

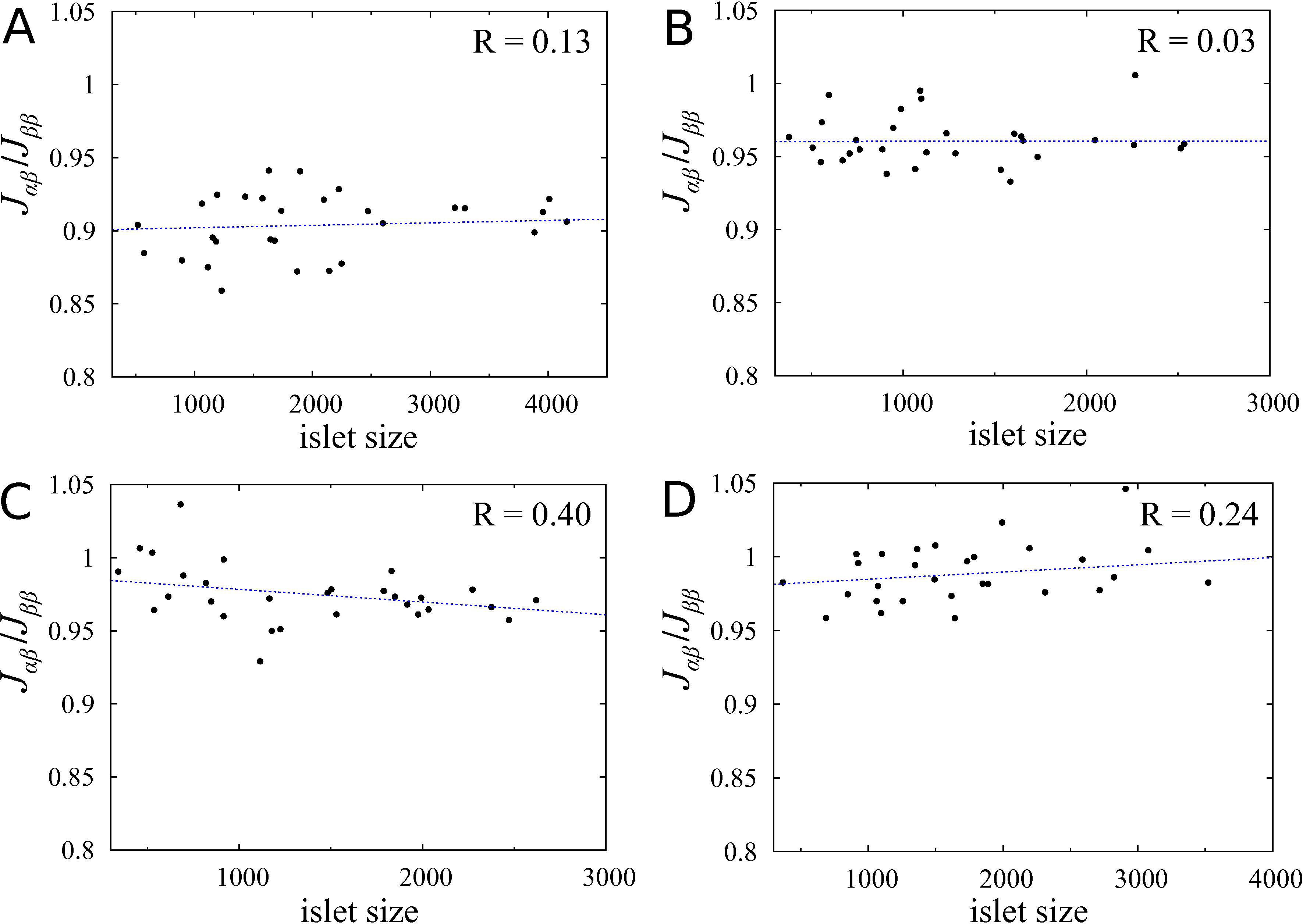

Supplement: Figure S1 — Relative cellular attractions and islet size. Relative attractions between cell types are inferred from three-dimensional islet structures in (A) mouse, (B) pig, and two human, (C) Human1 and (D) Human2, subjects. Islet size is represented by the total number of cells in islets. Symbols represent individual islets, and lines represent linear data fits (dotted blue). Here the linear regression analysis rejects the null hypothesis that the relative cellular attractions depend on islet size with high values for mouse (), pig (0.87), Human1 (0.03), and Human2 (0.20). values in the plots represent the coefficient of determination. Note that the attraction between cells is defined as a reference attraction, . Here thermal fluctuation energy is . (TIF) [file pone.0110384.s001.tif]

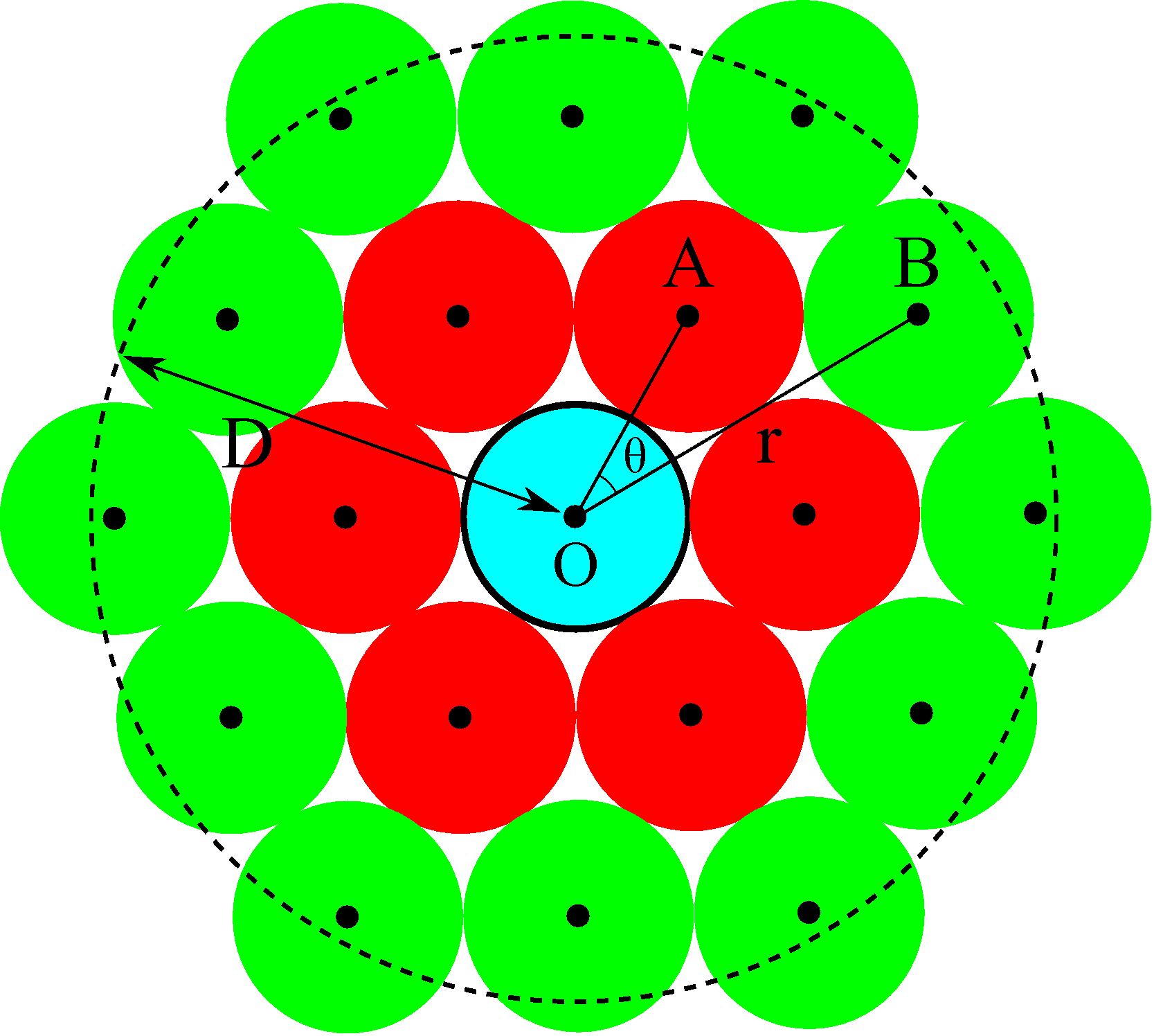

Supplement: Figure S2 — Determination of cellular contacts. Neighboring cells that contact to a given cell (cyan) are determined by cellular distance and angle . Sometimes second-nearest neighboring cells can be located within a threshold distance for determining neighbors. In the diagram, nearest-neighboring (red) and second-nearest neighboring (green) cells are defined as neighbors just based on distance (). However, once we include them, intercellular angles ( = AOB) between the neighbors can be smaller than an angle threshold . The angle threshold () can be used to further discriminate neighboring cells, close to the given cell (cyan), but not contacting. (TIF) [file pone.0110384.s002.tif]

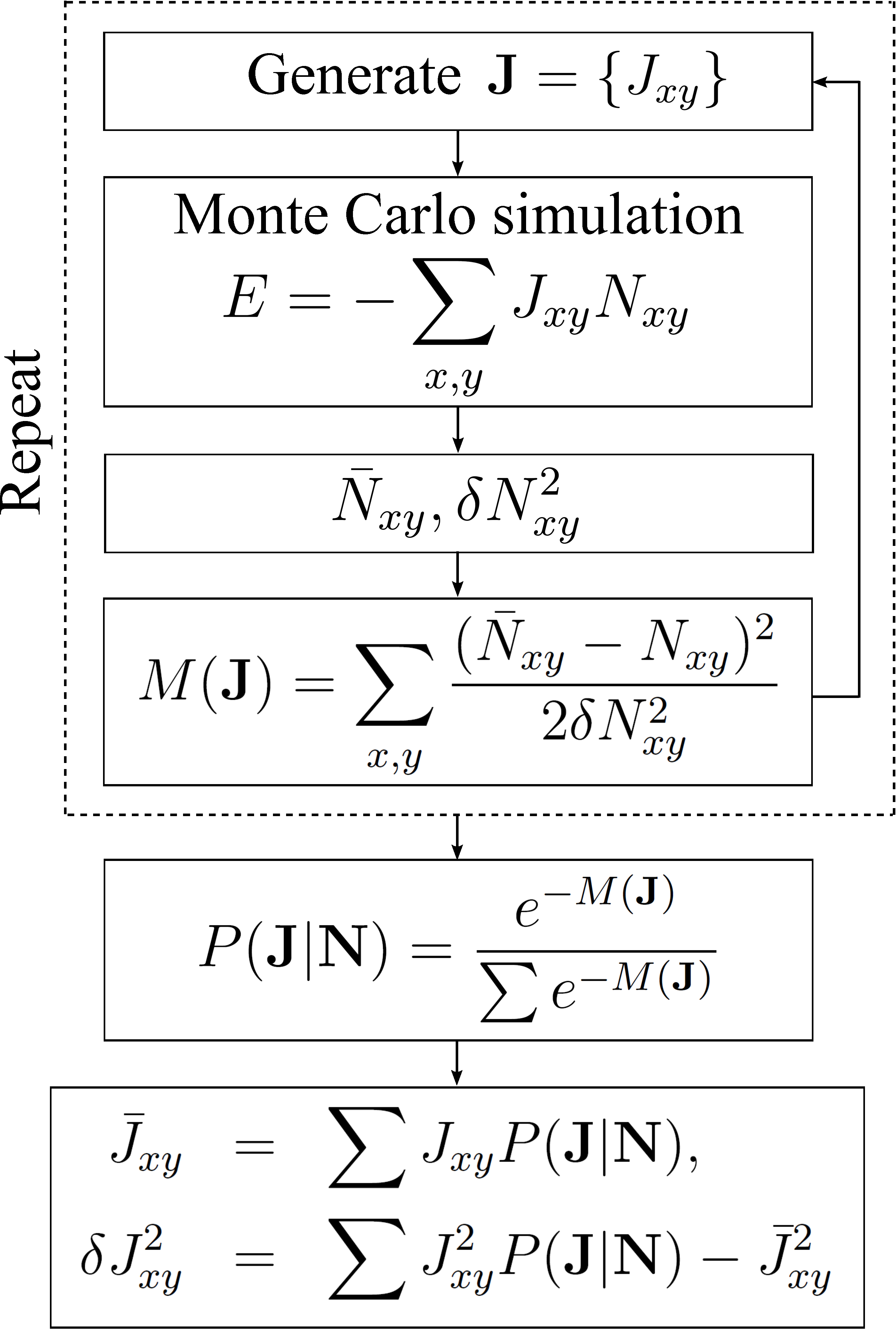

Supplement: Figure S3 — Flow chart for model equilibration and Bayesian inference. We generate cellular attraction energies, with , which are parameters of the differential adhesion model. Then, by using Monte-Carlo simulation, we equilibrate the islet self-energy, , given , where are the contact numbers between and cells. After equilibration, we obtain average numbers of cellular contacts , and their fluctuations . Finally, we compute the mismatch between predicted cellular contact numbers and the measured ones, . By repeating this procedure, we can have likelihood distribution of , given cellular contacts N = . This allows to estimate the likelihood and its uncertainty . (TIF) [file pone.0110384.s003.tif]

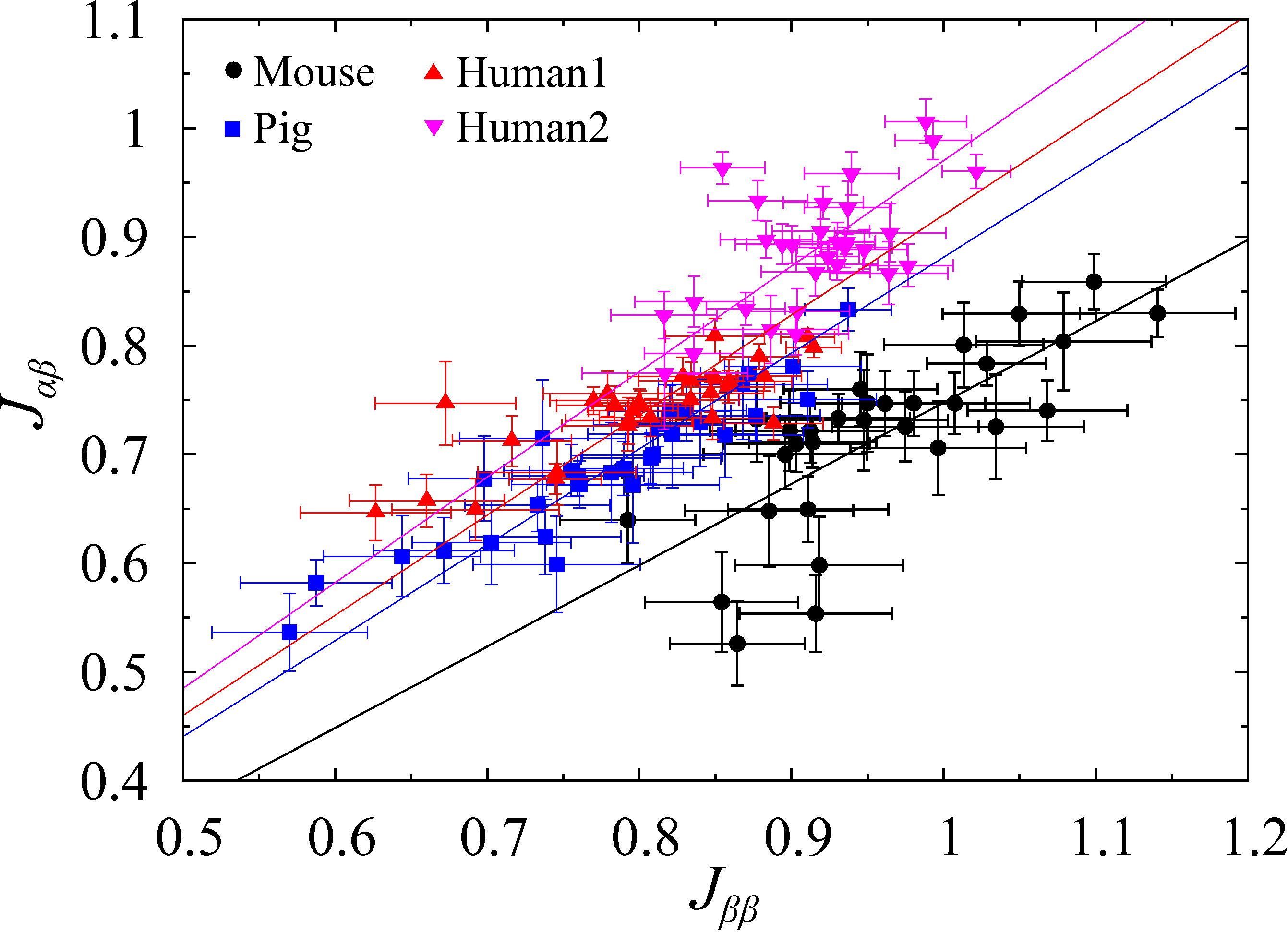

Supplement: Figure S4 — Cellular attractions at high thermal fluctuations. Relative attractions between cell types and their uncertainties are inferred from three-dimensional islet structures. Symbols represent individual islets: mouse (black circle), pig (blue square), and human islets (red triangle and pink inverse triangle). Each species has n = 30 islets. In particular, two sets of n = 30 islets are provided from two human (Human1 and Human2) subjects. The relationship between and is fitted with linear functions, , represented by solid lines with colors corresponding to each species. Note that the attraction between cells is defined as a reference attraction, . Here thermal fluctuation energy is . (TIF) [file pone.0110384.s004.tif]
