# Supplementary material for: A Conserved Rule for Pancreatic Islet Organization
Source: PLoS One. 2014 Oct 28;9(10):e110384. doi: 10.1371/journal.pone.0110384 (PMC4211668; doi:10.1371/journal.pone.0110384)
Supplement: Table S1 — Cellular attractions at high thermal fluctuations. (PDF) [file pone.0110384.s006.pdf]

Table S1. Cellular attractions at high thermal fluctuations

Species	n	$J_{\beta\beta}$	$J_{\alpha\beta}$	$J_{\alpha\beta}/J_{\beta\beta}$
Mouse	30	0.96 ± 0.08	0.72 ± 0.08^a	0.75 ± 0.06
Pig	30	0.78 ± 0.09	0.69 ± 0.07^a	0.88 ± 0.04^b
Human1	30	0.80 ± 0.07	0.74 ± 0.04^a	0.93 ± 0.06^c
Human2	30	0.91 ± 0.05	0.89 ± 0.06^a	0.97 ± 0.05^d

Relative attractions between cell types are inferred from three-dimensional islet structures, mean ± SD (n=30 islets). Note that the attraction between α cells is defined as a reference attraction, $J_{\alpha\alpha} = 1$. Here thermal fluctuation energy is $T = 0.5$. ^{a}Paired Student's t-test concludes $J_{\beta\beta} > J_{\alpha\beta}$ with $P < 0.005$. Unpaired Student's t-test concludes that $J_{\alpha\beta}/J_{\beta\beta}$ is different ^{b}between Mouse and Pig islets, ^{c}between Pig and Human1 islets, and ^{d}between Human1 and Human2 islets with $P < 0.005$.
